# Propionate metabolism-related molecular subtypes and prognostic signature in lung adenocarcinoma

**DOI:** 10.1097/MD.0000000000047270

**Published:** 2026-01-23

**Authors:** Zhifeng Li, Yuhui Cui, Yanchao Luan, Qingsong Han, Xuexiao Wang, Juan Li, Yuntao Nie, Liwei Yang

**Affiliations:** a Department of Thoracic Surgery, The Fourth Hospital of Hebei Medical University, Shijiazhuang, Hebei Province, China; b Department of Clinical Laboratory, Hebei General Hospital; Hebei Key Laboratory of Molecular Medicine; and Hebei Clinical Research Center for Laboratory Medicine, Shijiazhuang, Hebei Province, China; c Department of Thoracic Surgery, Hebei Chest Hospital, Shijiazhuang, Hebei Province, China; d Department of Radiation Oncology, The Fourth Hospital of Hebei Medical University, Shijiazhuang, Hebei Province, China; e Department of Medical Aesthetic, Xingtai People’s Hospital, Xingtai, Hebei Province, China.

**Keywords:** lung adenocarcinoma, molecular subtypes, prognostic signature, propionate metabolism, tumor microenvironment

## Abstract

Propionate exerts antiproliferative and immunomodulatory effects in tumors, but its role in the metabolism of lung adenocarcinoma (LUAD) remains underexplored. This study aimed to characterize molecular subtypes based on propionate metabolism-related genes (PMRGs) and assess their prognostic and immunological relevance in LUAD. Using transcriptomic data from The Cancer Genome Atlas (TCGA)–LUAD and validation from GSE30219, consensus clustering was performed to identify subtypes associated with propionate metabolism. Immune infiltration and tumor microenvironment characteristics were analyzed through established algorithms. Differentially expressed genes (DEGs) were identified, and a prognostic model was constructed using Cox and least absolute shrinkage and selection operator (LASSO) regression. Three molecular subtypes (low, medium, and high propionate metabolism) were identified, demonstrating significant differences in overall survival and immune microenvironment features. The high-propionate subtype was characterized by elevated immune and stromal scores, as well as increased M2 macrophage infiltration. A 7-gene prognostic signature was developed, with risk stratification revealing significant survival and drug sensitivity differences between high- and low-risk groups. Key prognostic genes, including *SLC2A1*, *SLC16A1*, *IL1A*, *AHSG*, and *ALOX15*, were validated through RT-qPCR. This study highlights the molecular heterogeneity of propionate metabolism in LUAD and proposes a prognostic signature that could inform immunotherapeutic stratification.

## 1. Introduction

Lung cancer remains the leading cause of cancer-related deaths globally, with the highest mortality rates observed in both men and women. In 2020, lung cancer claimed 1.8 million lives worldwide. In China alone, an estimated 8,20,100 new cases and 7,10,000 deaths occurred in 2020,^[1,2]^ accounting for 17.9% of new cancer cases and 23.8% of cancer-related deaths, respectively.^[3]^ Lung cancer is categorized into 2 main types: non-small cell lung cancer (NSCLC) and small cell lung cancer (SCLC). Lung adenocarcinoma (LUAD), the most prevalent form of NSCLC, represents over 60% of all cases. Advances in molecular targeted therapy and immunotherapy have significantly improved survival rates for LUAD; however, prognosis remains poor in advanced stages. In 2018, the 5-year survival rate for lung cancer was approximately 26.5%.^[4]^ Thus, identifying molecular targets and prognostic markers for LUAD is essential for predicting patient outcomes and monitoring disease progression.

Short-chain fatty acids (SCFAs), including acetate, propionate, and butyrate, are metabolites produced during the bacterial fermentation of dietary fiber in the gut.^[5,6]^ Recent research has increasingly focused on the role of SCFAs in cancer, with propionate emerging as a particularly promising compound due to its unique antitumor properties. Propionate regulates gene expression through histone acylation modifications, and its metabolic intermediates have been shown to impact mitochondrial function via a feedback loop, a mechanism critical in diseases such as lung cancer.^[7]^ Notably, Gomes et al demonstrated that dysregulated propionate metabolism enhances the invasive and metastatic potential of breast and lung cancer cells,^[8]^ suggesting that propionate metabolism contributes to tumor progression in a manner distinct from other SCFAs. Additionally, a study on the pathogenesis of tobacco-associated LUAD revealed that exposure to the tobacco-specific carcinogen NNK alters specific bacterial populations within the lung and gut microbiomes, leading to reduced SCFA levels, including propionate. These microbial changes are linked to the development of the LUAD phenotype and immune response.^[9]^ Together, these findings indicate that propionate metabolism plays a pivotal role in tumor microenvironment (TME) remodeling and malignant progression in LUAD, extending beyond its role as a typical SCFA. However, while much of the research has centered on butyrate, the role of propionate, especially in relation to the immune microenvironment and prognosis in LUAD, remains underexplored. This study aims to systematically investigate propionate metabolism in LUAD, identifying associated molecular subtypes, examining their relationship with prognosis and the immune microenvironment, and developing a robust prognostic model.

This study explored prognostic genes linked to propionate metabolism in LUAD and evaluated their prognostic significance using bioinformatics tools. A risk model for lung cancer prognosis was constructed based on propionate metabolism-related genes (PMRGs), demonstrating its stability and efficacy in prognostic evaluation.

## 2. Materials and methods

### 2.1. Access to and preprocessing of public data

The transcriptome sequencing dataset for LUAD was obtained from The Cancer Genome Atlas (TCGA) database (https://portal.gdc.cancer.gov/), which includes comprehensive mRNA expression profiles, clinical information, and mutagenic data. The TCGA–LUAD dataset consists of 513 tumor samples with survival information and 59 adjacent normal tissue samples (Tumor, n = 513; Normal, n = 59). Additionally, the GSE30219 dataset (microarray data) was retrieved from the Gene Expression Omnibus (GEO) database (http://www.ncbi.nlm.nih.gov/geo) to serve as an external cohort for model validation and expression analysis. This dataset includes 85 tumor samples with survival data and 14 normal tissue controls. A search using the “propionate metabolism” keyword in the GeneCards database (https://www.genecards.org/) with a relevance score > 7 identified 604 associated genes.^[10,11]^

### 2.2. Consensus clustering

Unsupervised clustering of LUAD samples from the TCGA–LUAD dataset was performed using the ConsensusClusterPlus package (version 1.58.0).^[12]^ Clustering was based on PMRGs, and the cumulative distribution function (CDF) plots were used to determine the optimal number of clusters by comparing the area changes in the CDF curves. The propionate metabolism scores of LUAD samples were then quantified using the ssGSEA algorithm, which helped identify molecular subtypes associated with propionate metabolism. Survival curves for each LUAD cohort were generated using the Survminer package (version 3.2-13).^[13]^ A heatmap depicting the expression of PMRGs across different subtypes was constructed with the pheatmap package (version 1.012).^[14]^

### 2.3. Immunoassay and somatic mutation analysis of different subtypes

To analyze the TME, the Cell-type Identification By Estimating Relative Subsets Of RNA Transcripts (CIBERSORT) algorithm was applied to estimate the abundance of 22 types of infiltrating immune cells from TCGA–LUAD RNA-seq data. The Estimation of STromal and Immune cells in MAlignant Tumor tissues using Expression data (ESTIMATE) method was used to assess stromal, immune, and estimate scores.^[15]^ Mutation analysis was conducted using the maftools package (version 2.10.5) on the TCGA–LUAD dataset.^[16]^

### 2.4. Differential expression analysis

Differentially expressed genes (DEGs) between the Tumor and Normal groups were identified using the DESeq2 package (version 1.34.0).^[17]^ The *P*-value was adjusted using the Benjamini-Hochberg method, and the criteria for DEG selection were |log_2_ fold change (FC)| > 1 and false discovery rate (FDR) < 0.05. These DEGs were related to propionate metabolism. Kaplan–Meier (KM) survival analysis of the molecular subtypes with the most significant survival differences was performed to identify DEGs with the same thresholds, referred to as subtype differential genes. The intersection of the propionate metabolism-related DEGs and the subtype differential genes with PMRGs was used to select candidate genes. Biological functional analysis^[18]^ was carried out using the clusterProfiler package (version 4.0.5), including Gene Ontology (GO) and Kyoto Encyclopedia of Genes and Genomes (KEGG) pathways, with significance defined as *P* < .05.^[19,20]^

### 2.5. Development of prognostic signature based on PMRGs

Following the identification of PMRGs with significant prognostic impact using univariate Cox regression analysis (*P* < .05), LASSO regression was performed to determine the precise coefficient values for each identified association. The risk score for each LUAD sample in the TCGA–LUAD dataset was calculated using the formula: risk score = $\;\;\;\underset{i=1}{\overset{n}{\sum }}\;\;\beta \;i*xi$, where β_*i*_ represents the regression coefficient and *x_i_* denotes the expression value of each gene. LUAD samples were then categorized into low-risk and high-risk groups based on the median risk score. KM survival curves were generated for both risk groups, and receiver operating characteristic (ROC) analysis was conducted using the Survminer package to assess the predictive performance of the model. The model was further validated using the external GSE30219 dataset.

### 2.6. Independent prognostic analysis and establishment of a nomogram

To identify independent prognostic factors, both univariate and multivariate Cox regression analyses were performed, incorporating risk scores along with clinical factors such as age, gender, T stage, N stage, and M stage. Independent prognostic factors were then used to construct a nomogram with the rms package (version 6.2.0). Calibration curves were plotted to evaluate the accuracy of the nomogram,^[21]^ and decision curve analysis was performed to assess its clinical utility. Additionally, the differences in risk score distributions and KM survival were analyzed across clinical indicators.

### 2.7. Risk group differential gene identification and enrichment analysis

DEGs between the risk groups were identified using the DESeq2 package, following the same selection criteria as described previously. Volcano plots were generated to visualize the differential expression results, and heatmaps were created based on |log_2_ FC|. The top 10 genes involved in enrichment pathways were highlighted. GO and KEGG enrichment analyses for the DEGs were performed using the clusterProfiler package.

### 2.8. Drug sensitivity analysis

Finally, chemotherapy sensitivity was predicted for the high- and low-risk groups using the pRRophetic package (version 0.5).^[22]^ Estimated half-maximal inhibitory concentration (IC_50_) values for each chemotherapeutic agent were obtained through regression, using default parameters that included averaging the “battle” and duplicate gene expression to eliminate batch effects.

### 2.9. Reverse transcription quantitative PCR (RT-qPCR)

A total of 20 clinical samples, including LUAD tissues and corresponding adjacent normal tissues, were randomly selected for RT-qPCR analysis. The clinical and pathological characteristics of all samples are provided in Table S1, Supplemental Digital Content, https://links.lww.com/MD/R198. The experimental process involved extracting total RNA using TRIzol (Ambion, Austin) reagent according to the manufacturer’s instructions, followed by reverse transcription into cDNA using the SureScript First-Strand cDNA Synthesis Kit. RT-qPCR reactions were performed using 2 × Universal Blue SYBR Green qPCR Master Mix (Servicebio, Wuhan, China), with GAPDH serving as an internal control. The relative expression of target genes was calculated using the 2^−ΔΔCT^ method, and each reaction was performed in triplicate. Primer sequences for the key genes and internal reference (GAPDH) are listed in Table S2, Supplemental Digital Content, https://links.lww.com/MD/R198.

### 2.10. Statistical analysis

A systematic analysis was performed using R software (version 4.1.0). ^[23]^First, unsupervised consensus clustering of the TCGA–LUAD cohort was conducted using the ConsensusClusterPlus package (version 1.58.0), which identified 3 molecular subtypes related to propionate metabolism. Differential expression analysis was then carried out using DESeq2 (version 1.34.0) to identify key genes. For TME analysis, the ESTIMATE algorithm was used to evaluate stromal and immune components. The somatic mutation landscape and oncogenic pathways across different subtypes were analyzed using maftools (version 2.10.5). Additionally, univariate Cox regression and LASSO regression (glmnet package, version 4.1-2) were applied to identify key prognostic genes and construct a risk score model. The model’s predictive performance was assessed using KM curves and ROC analysis. A prognostic nomogram was subsequently developed by integrating clinicopathological features (rms package, version 6.2-0), and drug sensitivity differences were analyzed using pRRophetic (version 0.5). Differences between groups were analyzed using the Wilcoxon test or *t*-test, and correlations among variables were examined using Pearson or Spearman correlation tests. For all statistical tests, 2-sided *P*-values of <.05 were considered statistically significant.

## 3. Results

### 3.1. Identification of LUAD subtypes in propionate metabolism

Unsupervised clustering was performed on LUAD samples from the TCGA–LUAD dataset based on PMRGs. The optimal number of clusters was determined by evaluating the relative changes in the CDF curves and their areas for *k* = 2–9. The results indicated that *k* = 3 yielded the most distinct clustering, as evidenced by the rapid decrease in the CDF curve and clear differentiation in the matrix heatmap,^[24]^ which distinctly displayed blue and white colors. Consequently, the samples were categorized into 3 clusters: cluster 1, cluster 2, and cluster 3 (Fig. S1A–D, Supplemental Digital Content, https://links.lww.com/MD/R199). These clusters were further classified into low (cluster 2), medium (cluster 1), and high (cluster 3) propionate metabolism subtypes based on the propionate metabolism scores (Fig. S1E, Supplemental Digital Content, https://links.lww.com/MD/R199).

### 3.2. Prognostic and clinicopathologic characterization of different subtypes

**Figure 1.**
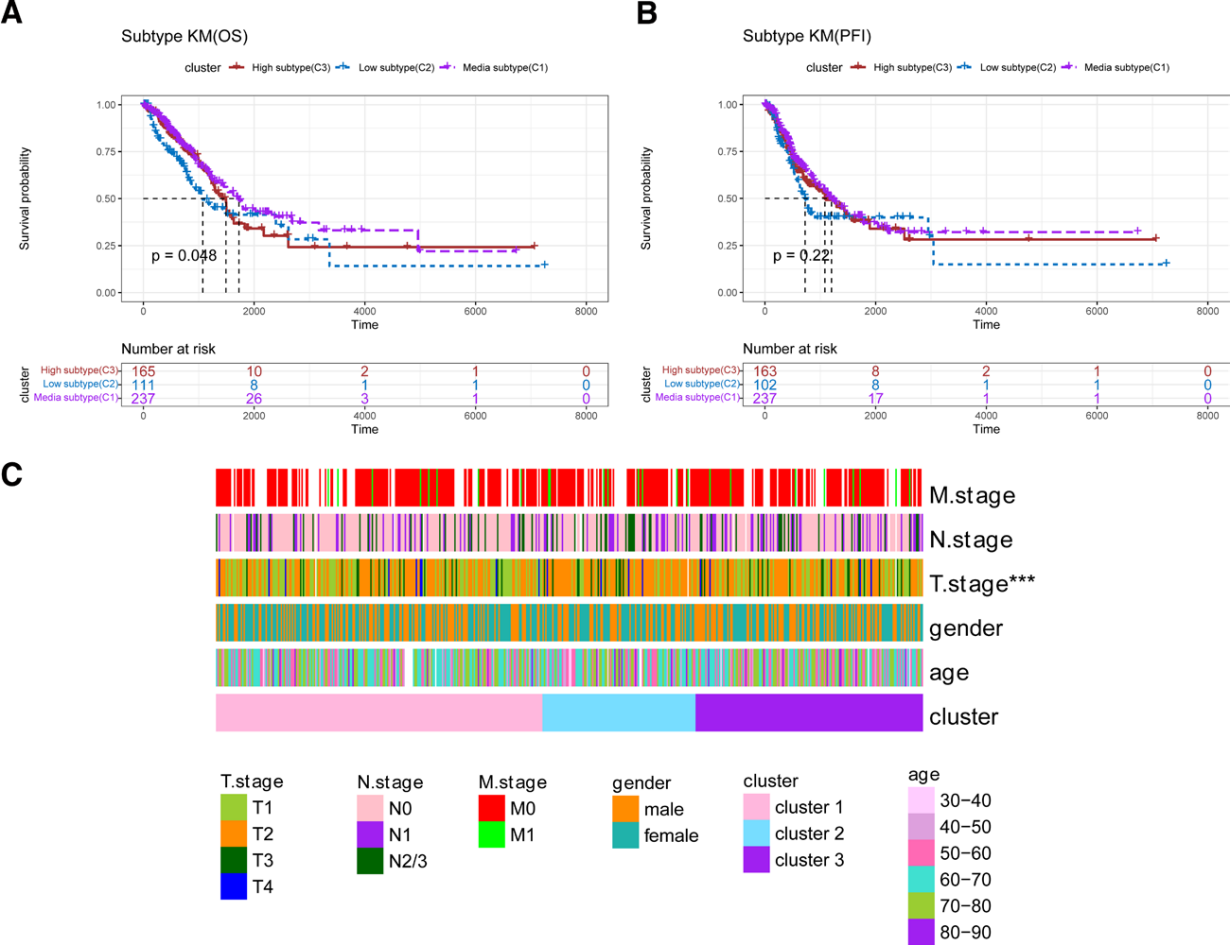
Survival analysis of Kaplan–Meier (KM) by molecular subtypes. (A) KM curves for overall survival of LUAD patients based on 3 molecular subtypes: high propionate metabolism (cluster 3), medium propionate metabolism (cluster 1), and low propionate metabolism (cluster 2). (B) KM curves for relapse-free survival in LUAD patients. (C) Expression patterns of 3 molecular subtypes across different clinical subgroups of LUAD. KM = Kaplan–Meier, LUAD = lung adenocarcinoma, progression-free interval.

Survival analysis of the molecular subtypes revealed a statistically significant difference in overall survival among the 3 subtypes (*P* < .05), while no significant difference was observed in progression-free interval (PFI; Fig. 1A, B). Heatmaps illustrating the distribution of clinicopathological features (age, gender, T, N, and M staging) across the subtypes highlighted significant differences in T staging (*P* < .05; Fig. 1C). These results suggest that molecular subtypes defined by propionate metabolism in LUAD hold diagnostic value for predicting LUAD prognosis.

### 3.3. Correlation of LUAD molecular subtypes with the immune status

**Figure 2.**
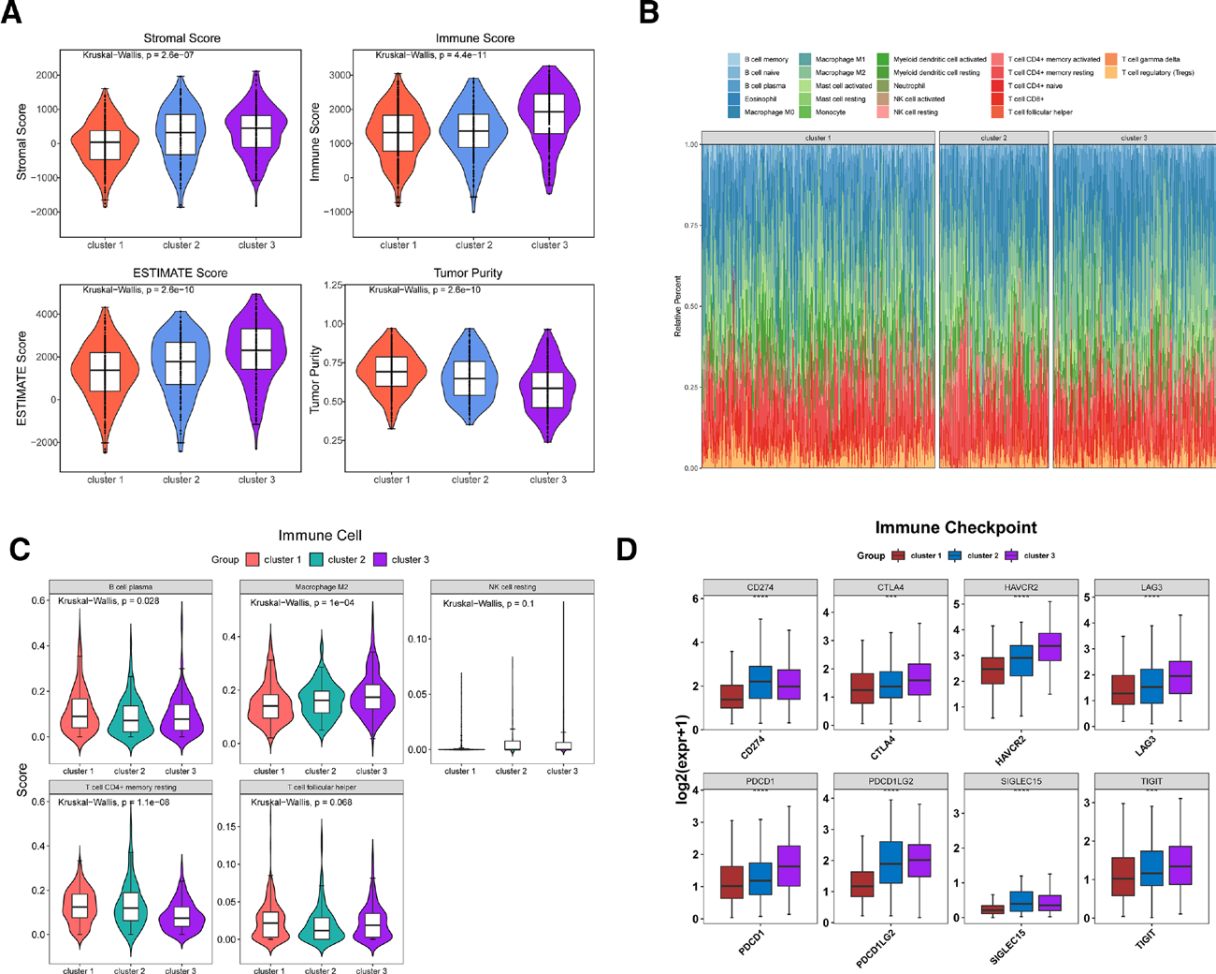
Analysis of tumor microenvironment in different molecular subtypes. (A) Immune score, stromal score, ESTIMATE score, and tumor purity across different molecular subtypes of propionate metabolism in LUAD. Orange represents cluster 1, blue represents cluster 2, and purple represents cluster 3. (B) Infiltration patterns of the 3 molecular subtypes across 22 immune cell types. The vertical axis represents the proportion of each cell type, and the horizontal axis represents the clusters. (C) Distribution differences of the 3 molecular subtypes in immunosuppressive cells. (D) Expression differences of the 3 molecular subtypes across 8 immune checkpoints. ***P < .001, ****P < .0001. ESTIMATE = estimation of stromal and immune cells in malignant tumors using expression data, LUAD = lung adenocarcinoma.

In this study, the immune profiles of the 3 subtypes were analyzed. The ESTIMATE algorithm was employed to evaluate the presence of stromal and immune cells in malignant tumor tissues based on expression data. The ESTIMATE analysis revealed significant differences in immunity scores, stromal scores, ESTIMATE scores, and tumor purity across the molecular subtypes. Notably, cluster 3 exhibited higher immune scores, stromal scores, and ESTIMATE scores, alongside the lowest tumor purity (Fig. 2A). These scores are strongly associated with immunotherapy outcomes, suggesting potential strategies for stratified immunotherapy targeting distinct molecular subtypes. Subsequently, immune cell infiltration profiles were analyzed using the CIBERSORT algorithm. Although the 22 immune cell infiltration profiles were similar across the 3 subtypes, significant differences were observed in the distribution of plasma B cells, M2 macrophages, and resting memory CD4+ T cells. M2 macrophages, which promote tumor growth and metastasis, were particularly abundant in cluster 3, indicating a potential for immune evasion in this subtype (Fig. 2B, C). Immune checkpoints are crucial targets for immunotherapy. The expression of 8 common immune checkpoints (PDCD1 (PD1), PDCD1LG2, CD274 (PD-L1), LAG3, TIGIT, CTLA4, HAVCR2 (TIM-3), and SIGLEC15) was examined across the 3 molecular subtypes. Remarkably, all 8 immune checkpoints showed significant expression differences between the subtypes. Cluster 3 exhibited the highest expression levels for all 8 checkpoints, suggesting a potentially lower efficacy of immune checkpoint inhibitor therapy in this group (Fig. 2D).

### 3.4. The status of somatic cell mutations and tumor microenvironment in different subtypes

**Figure 3.**
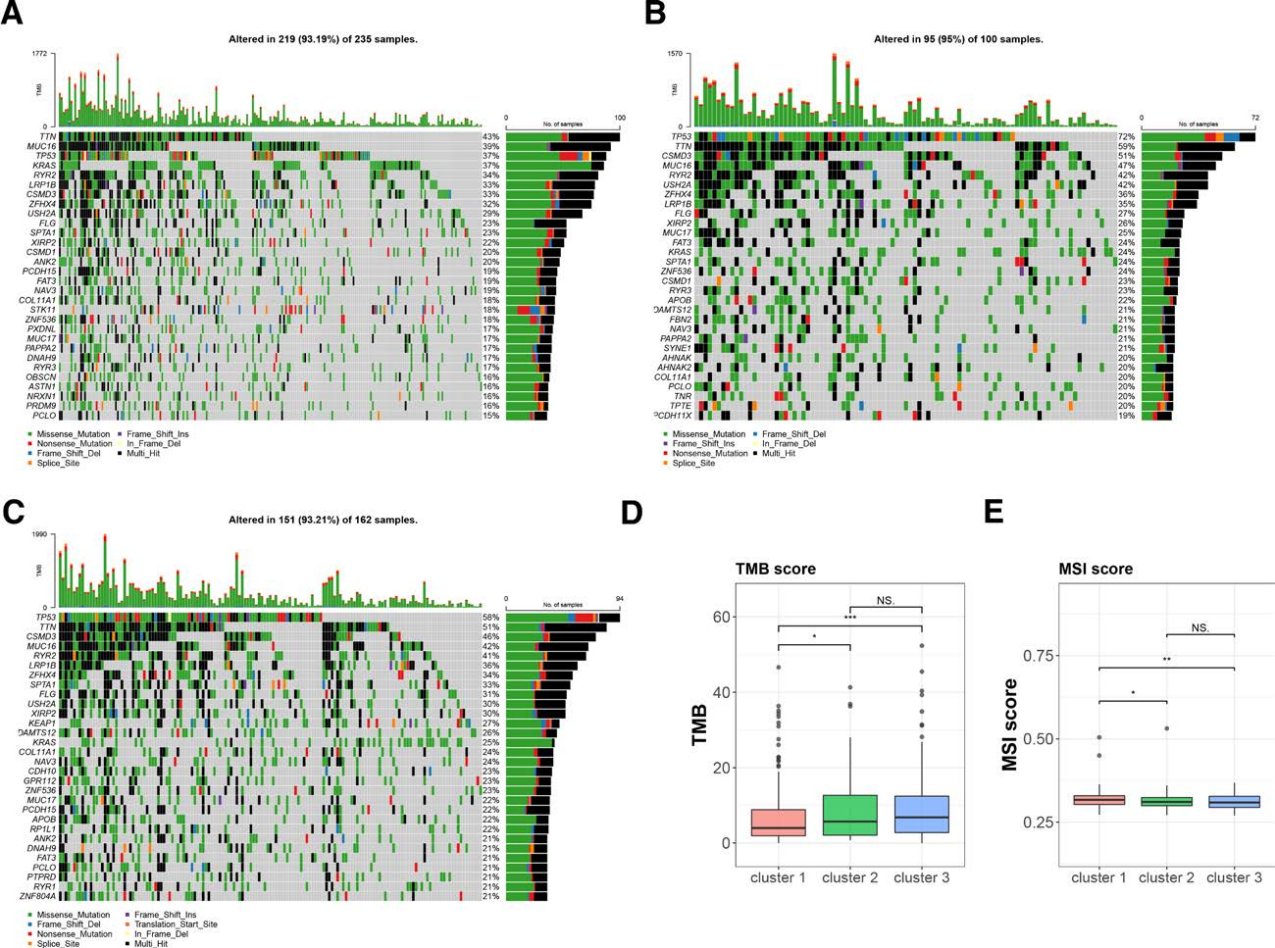
Somatic mutation status in different molecular subtypes. (A–C) Somatic mutations in the 3 molecular subtypes: (A) cluster 1, (B) cluster 2, (C) cluster 3. (D, E) Differences in tumor mutation burden (TMB) and microsatellite instability (MSI) scores among the 3 molecular subtypes. (F–H) Mutation frequencies in oncogenic pathways across the 3 molecular subtypes. MSI = microsatellite instability, TMB = tumor mutation burden.

To explore the genetic factors involved in tumorigenesis, somatic mutation profiles of the 3 molecular subtypes were analyzed, with the top 30 most commonly mutated genes visualized. TTN had the highest mutation frequency in cluster 1 (43%), while TP53 mutations were most frequent in clusters 2 and 3, at 72% and 58%, respectively. Cluster 2 had the highest somatic mutation rate at 95% (95 out of 100 samples), predominantly consisting of missense mutations (Fig. 3A–C). Tumor mutational burden (TMB), microsatellite instability (MSI), and neoantigens are known to influence antitumor immunity and predict the efficacy of immunotherapy. Significant differences in TMB and MSI scores were observed between cluster 1 and both cluster 2 and cluster 3, further supporting the notion that molecular subtypes modulate tumor immunity through alterations in the TME (Fig. 3D, E). Additionally, the frequency of mutations in major oncogenic pathways was assessed, revealing that the RTK-RAS, WNT, and NOTCH signaling pathways were most frequently implicated across the 3 subtypes (Fig. S2A–C, Supplemental Digital Content, https://links.lww.com/MD/R199).

### 3.5. Identification of DEGs based on LUAD molecular subtypes

**Figure 4.**
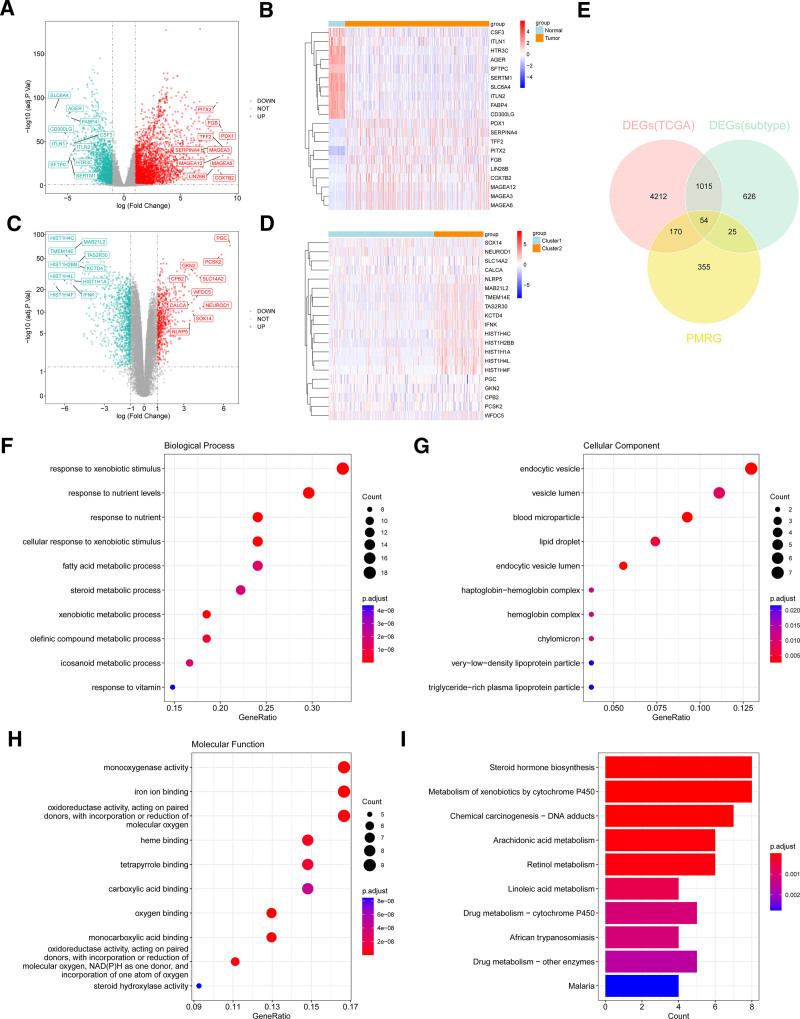
Identification of differentially expressed genes (DEGs) based on LUAD molecular subtypes. (A) Volcano plot of differentially expressed genes related to propionic acid metabolism in TCGA–LUAD. Green represents down-regulated genes, red represents up-regulated genes in LUAD samples, and gray represents genes with no significant differences. (B) Heatmap of differentially expressed genes related to propionic acid metabolism. Red indicates high expression, blue indicates low expression. (C) Volcano plot of subtype differential genes in molecular subtypes. Green represents down-regulated genes, red represents up-regulated genes in cluster 1, and gray represents genes with no significant differences. (D) Heatmap of subtype differential genes. Red indicates high expression, blue indicates low expression. (E) Candidate genes obtained by intersecting differentially expressed genes related to propionic acid metabolism and subtype differential genes with PMRG. (F–H) Functional enrichment results of candidate genes in GO categories: biological process, cellular component, and molecular function. (I) KEGG enrichment results. The closer the color to red, the more significantly enriched the pathway. GO = Gene Ontology, KEGG = Kyoto Encyclopedia of Genes and Genomes, PMRG = Propionate Metabolism-Related Genes, TCGA–LUAD = The Cancer Genome Atlas–Lung Adenocarcinoma.

A total of 5451 DEGs related to propionic acid metabolism were identified in the TCGA–LUAD dataset through differential expression analysis. Among these, 3488 genes were upregulated and 1963 genes were down-regulated. The expression levels of these DEGs are presented in Figure 4A, B. Based on the KM survival curves of different molecular subtypes, cluster 1 and cluster 2, which showed the largest survival differences, were selected for further analysis. A total of 1720 subtype-specific differential genes were identified, with 635 genes up-regulated and 1085 genes down-regulated in clusters 1 and 2 (Fig. 4C, D). These DEGs related to propionic acid metabolism and the subtype-specific differential genes were intersected with PMRGs, yielding 54 candidate genes (Fig. 4E). GO and KEGG analyses were performed to explore the potential functional pathways of these candidate genes (*P* < .05). GO enrichment analysis revealed that the candidate genes were involved in fatty acid metabolism, eicosanoid metabolism, organic acid binding, carboxylic acid binding, tetrapyrrole binding, and steroid hydroxylase activity. Propionate metabolism was specifically linked to acid-catalyzed metabolic processes, such as those involving fatty acids, amino acids, sugars, and branched-chain amino acids. KEGG analysis highlighted similar pathways, with the 54 candidate genes primarily enriched in linoleic acid metabolism, drug metabolism via cytochrome P450, and arachidonic acid metabolism. Notably, cytochrome P450 family members are known to be closely associated with LUAD development^[25]^ (Fig. 4F–I). These candidate genes were implicated in pathways related to both LUAD progression and propionate metabolism.

### 3.6. Construction of a propionate metabolism-related risk model

**Figure 5.**
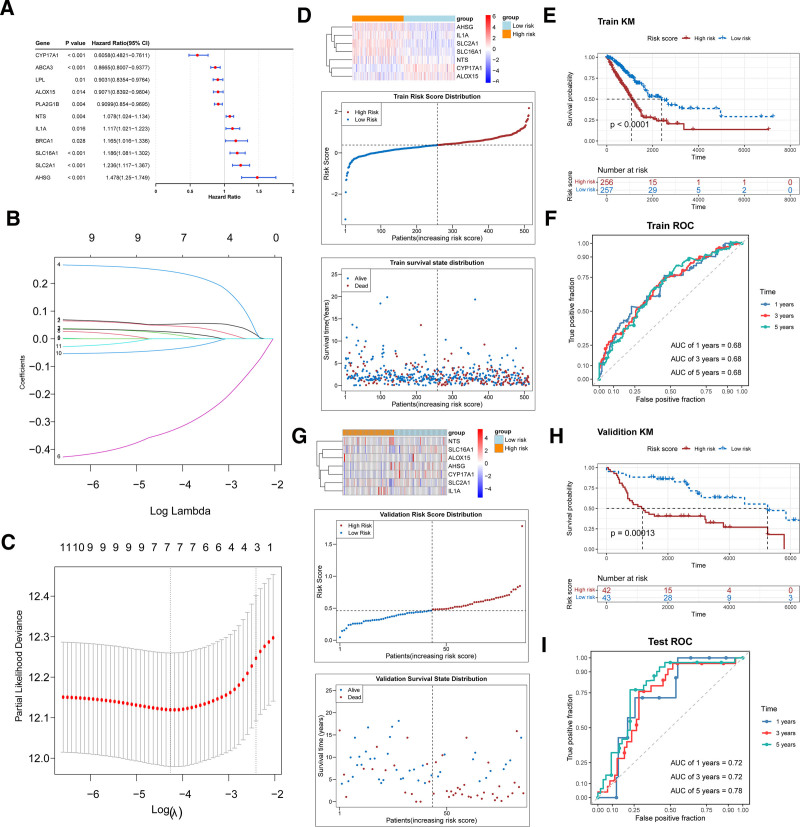
Construction and validation of a risk model related to propionate metabolism. (A) Forest plot of univariate Cox regression analysis. (B) Variable selection and coefficient shrinkage via LASSO regression. The *x*-axis represents log(λ) values, the *y*-axis shows regression coefficients, and the colored lines track how the coefficients of different genes change as log(λ) varies. (C) Cross-validation results of LASSO regression. The *x*-axis represents log(λ) values, the *y*-axis shows partial likelihood deviance, and the red dots indicate changes in partial likelihood deviance with log(λ). The left dashed line marks the position of λ _min_, and the right dashed line indicates λ _1_SE. (D) From top to bottom: expression patterns of prognostic genes in the risk groups, the division of risk scores, and the distribution of LUAD samples across the risk groups. (E) Survival differences between LUAD patients in the TCGA–LUAD dataset in high- and low-risk groups. The *x*-axis represents follow-up time (d), the *y*-axis indicates survival probability, and the red and blue curves correspond to the high-risk and low-risk groups, respectively. (F) Kaplan–Meier survival curves and 1-, 3-, and 5-year ROC curves in the TCGA–LUAD dataset. (G) Survival differences between LUAD patients in the GSE30219 dataset in high- and low-risk groups. (H) Survival differences between LUAD patients in the GSE30219 dataset in high- and low-risk groups. (I) Kaplan–Meier survival curves and 1-, 3-, and 5-year ROC curves in the GSE30219 dataset. LASSO = least absolute shrinkage and selection operator, LUAD = lung adenocarcinoma, TCGA–LUAD = The Cancer Genome Atlas–Lung Adenocarcinoma, ROC = receiver operating characteristic.

**Figure 6.**
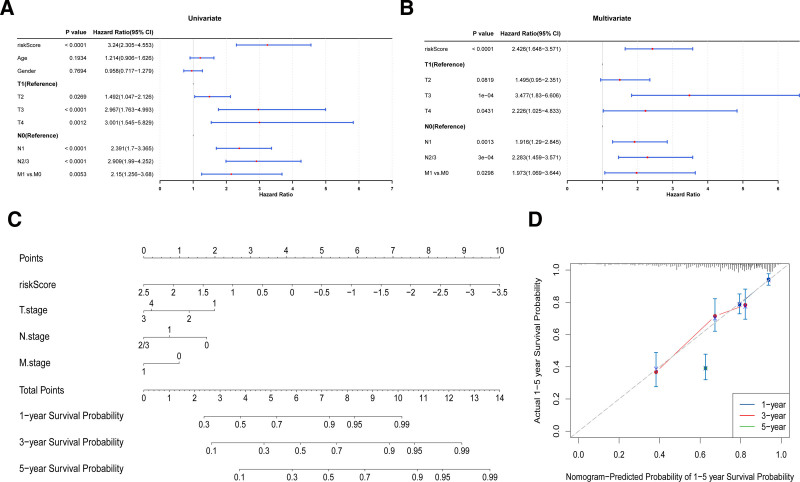
Independent prognostic analysis and nomogram construction. (A) Univariate Cox regression analysis of risk scores and clinical indicators. (B) Multivariate Cox regression analysis. (C) Nomogram predicting the probability of survival at 1, 3, and 5 years. (D) Calibration curve to estimate survival probability at 1, 3, and 5 years. The horizontal axis shows the predicted probability of survival (1–5 years), and the vertical axis shows the actual survival probability (1–5 years).

Univariate Cox regression analysis of the 54 candidate genes identified 11 genes associated with prognosis (HR ≠ 1, *P* < .05), as shown in the forest plot (Fig. 5A). After performing least absolute shrinkage and selection operator (LASSO) regression analysis, 7 prognostic genes were identified: *SLC2A1*, *SLC16A1*, *IL1A*, *AHSG*, *CYP17A1*, *NTS*, and *ALOX15* (Fig. 5B, C). A risk model was constructed by calculating risk scores based on these prognostic genes, categorizing LUAD samples into high-risk (n = 256) and low-risk (n = 257) groups based on the median risk score (0.3814068). The distribution of risk scores and survival status is shown in Figure 5D. Notably, LUAD samples in the high-risk group had a significantly lower survival rate. This was further confirmed by the KM survival curve, which indicated that high-risk samples had markedly reduced survival (*P* < .0001). The predictive performance of the risk model was assessed using ROC curve analysis, and the ROC curves for 1, 3, and 5 years demonstrated that the model had good predictive ability, with AUC values above 0.6 (Fig. 5E, F). The model was further validated in the GSE30219 validation cohort, showing strong forecasting capability (Fig. 6G–I). Additionally, the expression levels of the prognostic genes in tumor and adjacent tissues from the TCGA–LUAD and GSE30219 datasets were compared. The expression patterns of these genes were consistent between the 2 datasets, although the difference in expression for *CYP17A1* and *NTS* did not reach statistical significance in the GSE30219 dataset (Figs. S3 and S4, Supplemental Digital Content, https://links.lww.com/MD/R199).

### 3.7. Independent prognostic analysis and construction of a nomogram

Univariate and multivariate regression analyses identified the risk score, T stage, N stage, and M stage as independent prognostic factors for the nomogram construction. The nomogram demonstrated a progressive increase in 1-, 3-, and 5-year survival rates for LUAD patients with higher total points. Calibration curve analysis confirmed the accuracy of the nomogram, with the curves at all 3 time points closely aligning with the reference line (Fig. 6A–D). This not only introduces a novel independent prognostic factor for LUAD but also provides a valuable decision-making tool for clinical management.

### 3.8. Analysis of risk score correlation with clinical features

KM survival curves for high- and low-risk groups were analyzed according to clinical indicators in different subgroups. The results revealed significant survival differences related to age, gender, T2/3, T3/4, N0, and M0 stages (*P* < .05;Fig. S5A, B, Supplemental Digital Content, https://links.lww.com/MD/R199). These results suggest that the risk score based on propionate metabolism features holds diagnostic potential for LUAD’s clinical characteristics.

### 3.9. Risk score-related DEGs and their functional characterization

To further investigate the differences between the risk groups, differential expression analysis identified a total of 1442 DEGs. Among these, 700 genes were up-regulated in the high-risk group, while 742 genes were specifically up-regulated in the low-risk group. The expression levels of these DEGs are shown in Fig. S6A, B, Supplemental Digital Content, https://links.lww.com/MD/R199. GO enrichment analysis revealed that the DEGs were significantly associated with movement-related pathways, such as cilium movement, cilium or flagellum-dependent cell motility, and cilium movement involved in cell motility. Ciliogenesis has been shown to activate the Hh signaling pathway, which is associated with a decreased survival probability in LUAD patients. Additionally, KEGG pathway analysis highlighted significant enrichment in pathways related to the metabolism of xenobiotics by cytochrome P450, drug metabolism-cytochrome P450, and steroid hormone biosynthesis (Fig. S6C, D, Supplemental Digital Content, https://links.lww.com/MD/R199).

### 3.10. Drugs with potential efficacy in LUAD

The study also examined the predictive potential of 138 chemotherapeutic/targeted therapeutic agents. Using the pRRophetic algorithm, the IC_50_ values for these drugs were estimated. Notably, 118 drugs showed statistically significant differences between the risk groups (*P* < .05). Among these, 55 drugs had a lower IC_50_ in the high-risk group compared to the low-risk group, with dasatinib and erlotinib being the most commonly targeted. Conversely, the IC_50_ of the common blockbuster drug sunitinib was higher in the high-risk group (Fig. S7A, B, Supplemental Digital Content, https://links.lww.com/MD/R199). These findings highlight the importance of risk-stratified treatment for LUAD, potentially improving drug efficacy for patients.

### 3.11. The expression levels of the prognosis biomarkers

**Figure 7.**
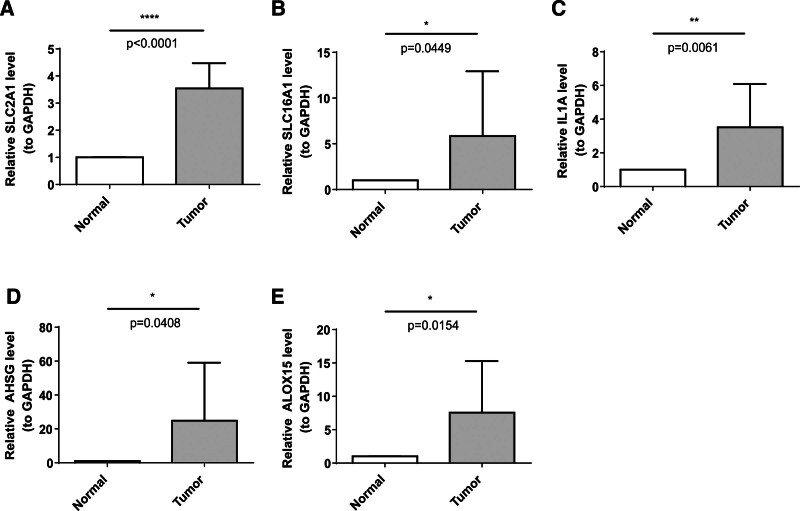
RT-qPCR analysis of prognostic gene expression levels in clinical samples. (A) *SLC2A1*, (B) *SLC16A1*, (C) *IL1A*, (D) *AHSG*, and (E) *ALOX15 (GAPDH* as internal reference gene, *P < .05). RT-qPCR = reverse transcription quantitative PCR.

RT-qPCR results confirmed that the expression levels of the prognostic genes *SLC2A1*, *SLC16A1*, *IL1A*, *AHSG*, and *ALOX15* were significantly elevated in LUAD samples compared to normal tissues (Fig. 7A–E). However, some discrepancies were observed between the expression data and the dataset chosen, likely due to the limited number of clinical samples.

## 4. Discussion

LUAD, a subtype of non-small cell carcinoma, is the most prevalent histological form of lung cancer, characterized by high morbidity and mortality. Despite the availability of comprehensive treatments, including surgery, chemotherapy, targeted therapy, and immunotherapy, LUAD remains associated with poor prognosis due to local recurrence or distant metastasis.^[26]^ There is an urgent need to develop more reliable strategies for subtype identification and prognostic classification. The metabolic heterogeneity of LUAD has been widely acknowledged as a critical factor influencing tumor progression and treatment response. Previous studies have shown that focusing on specific metabolic pathways, such as glycolysis and glutaminolysis, can effectively identify molecular subtypes with significant prognostic value.^[27]^ Propionate, an SCFA, has been implicated in various cancers,^[28,29]^ but research on its role in LUAD remains limited compared to butyrate. This study aimed to investigate the relationship between propionate metabolism subtypes, LUAD prognosis, and their potential connections with the immune microenvironment.

Consensus clustering, an unsupervised method for identifying molecular subtypes based on gene expression data,^[30]^ was employed in this study. Three LUAD subtypes were identified for the first time: the low propionate metabolism subtype (cluster 2), the medium propionate metabolism subtype (cluster 1), and the high propionate metabolism subtype (cluster 3), based on PMRGs from the TCGA–LUAD dataset. Prognostic and clinicopathologic feature analysis revealed significant differences in overall survival and T stage among the 3 subtypes. The findings highlight that these subtypes influence tumor pathological staging, particularly the T stage, and could serve as potential predictors of tumor progression. This research enhances our understanding of the role of propionate metabolism in LUAD development.

The TME plays a pivotal role in tumor progression, encompassing stromal cells, immune cells, tumor cells, and other biological factors. Within the TME, immune cells are often densely concentrated around and within the tumor, with intricate interactions between immune cells themselves and between tumor and immune cells.^[31]^ Our findings revealed significant distribution differences among the 3 molecular subtypes for M2 macrophages, CD4+ T cells, and B cells. A study by Sedighzadeh et al^[32]^ demonstrated that M2 macrophages are typically found in environments associated with type 2 helper T cells (Th2). Common markers for M2 macrophages, such as arginase, CD206, CD204, and CD163, are well-suited for inflammatory response inhibition and tumor progression, positioning them as promising therapeutic targets for lung cancer treatment. Furthermore, Veatch et al^[33]^ highlighted the critical role of CD4+ class II MHC-restricted T cells in antitumor immunity. Tumor metastasis, a primary factor in poor prognosis for LUAD patients, has been shown to be facilitated by tumor-associated macrophages (TAMs), which promote epithelial-mesenchymal transition (EMT) and metastasis through a positive feedback loop involving IL6-STAT3-C/EBPβ-IL6.^[34]^ Tumor-infiltrating B lymphocytes (TIBs), key components of the TME, play a vital role in tumor development across all cancer stages.^[35]^ Targeting TIBs may offer novel therapeutic strategies for NSCLC patients.

Immune checkpoints, which include a range of molecules expressed on immune cells, regulate immune activation by preventing excessive immune responses and maintaining balance within the immune system.^[36]^ The study revealed differential expression of immune checkpoints, including PD-L1 (CD274), CTLA4, TIM-3 (HAVCR2), LAG3, PD1 (PDCD1), PDCD1LG2, TIGIT, and SIGLEC15, across the 3 molecular subtypes. Previous research has shown that monoclonal antibodies targeting PD-1 and its ligands, as well as CTLA4, have been approved for clinical use in NSCLC therapy.^[37]^ Among these, PD-L1 (CD274), CTLA4, LAG3, PD1 (PDCD1), and TIGIT have been associated with lung cancer, although the precise mechanisms underlying their role in tumor progression remain unclear. The abnormal infiltration of immune cells and differential expression of immune checkpoints offer valuable prognostic indicators and therapeutic targets for immunotherapy, highlighting their clinical significance.

Stromal-mediated and immune-high profiles are associated with favorable outcomes in LUAD.^[38]^ In the present study, molecular subtypes with medium and high propionate metabolism (clusters 2 and 3) exhibited significantly higher survival rates compared to the low propionate metabolism subtype. Notably, cluster 3 showed a predominant distribution of M2 macrophages and achieved the highest scores in immune, stromal, and ESTIMATE assessments. Additionally, cluster 3 demonstrated elevated expression of 8 common immune checkpoints. These findings suggest that high propionate metabolism may be linked to a favorable prognosis in LUAD, while also potentially reducing the efficacy of immune checkpoint inhibitors. A previous study highlighted progressive changes in gut and lung microbiome members (e.g., Odoribacter, Alistipes, Akkermansia, and Ruminococcus), which were closely associated with the phenotypic development of LUAD and the response to immunotherapy in lung cancer patients. These changes, particularly a reduction in SCFAs (including propionic and butyric acids) following NNK exposure,^[39]^ further support the potential connection between propionate metabolism and immune regulation in LUAD. This provides preliminary theoretical evidence for the clinical utility of risk stratification strategies based on propionate metabolism in the precision treatment of LUAD.

In this study, 7 prognostic genes linked to LUAD were identified. Expression analysis from both the training and validation sets revealed 5 prognostic genes (*SLC2A1*, *SLC16A1*, *IL1A*, *AHSG*, and A*L*OX15) exhibiting consistent trends and significant differences. A risk model was then constructed based on these genes. Wang et al^[40]^ confirmed that *SLC2A1* promotes LUAD cell growth and migration by enhancing glucose utilization, with a significant correlation between *SLC2A1* expression and LUAD prognosis. In lung cancer, increased *SLC16A1* expression has been associated with poor prognosis in LUAD patients.^[41]^ *IL1A*, a highly pro-inflammatory cytokine, increases tumor angiogenesis and invasiveness within the TME. Li et al^[41]^ demonstrated that high *IL1A* levels in pleural effusions are linked to the invasion of NSCLC cells. The protein product of *AHSG*, Fetuin-A, is a multifunctional protein recognized for its role in cancer. Fetuin-A in the TME may promote efficient synthesis, secretion, and endocytosis of exosomes, thereby aiding tumor growth. High ectopic Fetuin-A expression in lung cancer correlates with lower survival rates.^[42]^ Moreover, inhibiting AHSG expression in LUAD cell lines suppressed proliferation, migration, and invasion while blocking the EMT process.^[43]^ These findings suggest that AHSG may be involved in LUAD cell migration. Reprogramming propionate metabolism is proposed to play a critical role in modulating responses to immunotherapy. A recent single-cell analysis also highlighted the impact of purine metabolism heterogeneity within the LUAD TME on patient prognosis and the efficacy of immunotherapeutic interventions.^[44]^ Consistently, *SLC2A1*, *SLC16A1*, and *AHSG* were significantly upregulated in LUAD samples from both datasets and clinical samples.

The role of *ALOX15* in carcinogenesis remains controversial, with both pro- and anticancer effects reported. Pro-cancer effects have been observed in prostate and colorectal cancers, while anticancer effects have been noted in lung, esophageal, and breast cancers.^[45]^ A study using a Lewis lung carcinoma model showed that endothelial cell-specific overexpression of *ALOX15* promotes apoptosis and necrosis in both primary and metastatic tumors by up-regulating P21 and PPARγ expression in neighboring cancer cells.^[46]^ The present study reveals the prognostic significance of propionate metabolism in LUAD, suggesting that the underlying mechanisms could harbor novel therapeutic targets and offer potential directions for developing targeted strategies against LUAD.

In the analysis of risk groups, a total of 118 drugs exhibited differential sensitivity, with 55 drugs showing higher IC_50_ values in the low-risk group and 63 drugs exhibiting higher IC_50_ values in the high-risk group. Among these, several agents are specifically indicated for treating lung cancer and its subtypes, including Pemetrexed, Gemcitabine, Paclitaxel, Cisplatin, Lapatinib, and Sorafenib.^[47,48]^ Drug resistance has been recognized as a key factor influencing therapeutic efficacy in LUAD patients. Despite significant progress in understanding drug resistance mechanisms^[49,50]^ and combination therapy,^[47]^ the availability of effective drugs and their substantial therapeutic benefits remain central objectives. Our research suggests that constructing a reliable LUAD risk model based on propionate metabolism-related risk scores holds significant potential for risk-stratified treatment, offering new insights to reduce treatment resistance and potentially improve clinical outcomes for LUAD patients.

This study provides a comprehensive analysis of the prognostic and immunological significance of propionate metabolism in LUAD. However, several limitations should be noted. The primary findings are based on retrospective analyses of public databases and lack external validation from prospective clinical cohorts. The sample size for RT-qPCR validation was small (n = 20), limiting statistical power. Although the research identifies associations, causal relationships and specific mechanisms linking propionate metabolism to LUAD progression remain to be confirmed through functional experiments. Furthermore, the association between the propionate metabolism signature and drug sensitivity has not been experimentally validated, limiting its relevance for informing treatment strategies. Additional confounding factors, such as smoking status and TMB, may also influence the accuracy of the analytical results.

To overcome these limitations, future studies should focus on several key areas: utilizing single-cell sequencing technologies to analyze cell-type-specific expression patterns of propionate metabolism within the TME; validating the biological functions of key metabolic genes and associated metabolites through in vitro and in vivo experiments; conducting drug sensitivity assays to explore the relationship between the propionate metabolism signature and responses to chemotherapy, targeted therapy, or immunotherapy; and developing more robust prognostic models by integrating clinicopathological features with other metabolic subtypes across multicenter prospective cohorts. These efforts will enable a comprehensive evaluation of the clinical value of propionate metabolism in risk stratification and personalized treatment for LUAD.

## 5. Conclusions

This study employed the pRRophetic algorithm to evaluate the IC_50_ of patients in high-risk and low-risk groups from the TCGA dataset, revealing that the IC_50_ values for the high-risk group were lower for drugs such as dasatinib and erlotinib compared to the low-risk group, while the IC_50_ for sunitinib was higher in the high-risk group. These findings suggest potential therapeutic options for LUAD.

For the first time, 3 LUAD subtypes associated with propionate metabolism were identified in this study, and 5 prognostic genes were screened. A risk model based on PMRGs was constructed, showing a strong association with the tumor immune microenvironment and LUAD prognosis. These results suggest that propionate metabolism is potentially clinically relevant to the onset, progression, and prognosis of LUAD. This study examined the effects of propionate metabolism on the immune microenvironment, immune checkpoints, mutated genes, and drug sensitivity in LUAD. The identification of 5 novel genetic markers offers new prognostic insights for LUAD. After further validation, this research could contribute to the development of precision, stratified treatment strategies for LUAD.

## Acknowledgments

We would like to express our sincere gratitude to all individuals and organizations who supported and assisted us throughout this research. In conclusion, we extend our thanks to everyone who has supported and assisted us along the way. Without your support, this research would not have been possible.

## Author contributions

**Data curation:** Yuhui Cui, Yanchao Luan, Qingsong Han, Xuexiao Wang, Juan Li, Yuntao Nie.

**Formal analysis:** Yuhui Cui, Yanchao Luan, Qingsong Han, Xuexiao Wang, Juan Li, Yuntao Nie, Liwei Yang.

**Funding acquisition:** Liwei Yang.

**Writing – original draft:** Zhifeng Li.

**Writing – review & editing:** Liwei Yang.

## Supplementary Material




