# Supplementary material for: Propionate metabolism-related molecular subtypes and prognostic signature in lung adenocarcinoma
Source: Medicine (Baltimore). 2026 Jan 23;105(4):e47270. doi: 10.1097/MD.0000000000047270 (PMC12851720; doi:10.1097/MD.0000000000047270)

Supplementary Fig. S1 Consistent clustering results and ssGSEA score for the three molecular subtypes. (a) CDF curve distribution of consistent clustering for classification numbers k = 2-9. (b) Area distribution under the CDF curve for consistent clustering when classification number k = 2-9. (c) Clustering outcomes when the classification number k = 3. (d) Heatmap of expression for the three isoforms of propionate classification. (e) Differential analysis based on ssGSEA scores for the three molecular subtypes. ****P* < 0.001.

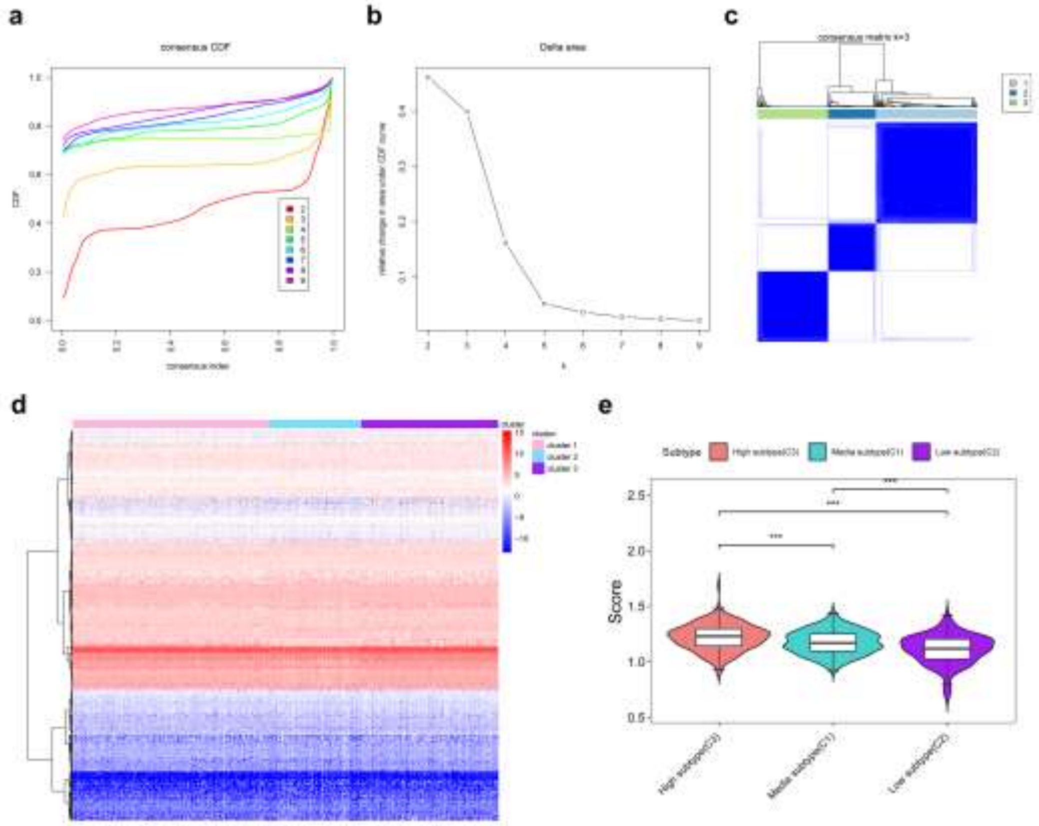

Supplementary Fig. S2 Main carcinogenic pathways of different subtypes. (a) Mutation frequency in the Cluster 1 oncogenic pathway. (b) Mutation frequency in the Cluster 2 oncogenic pathway. (c) Mutation frequency in the Cluster 3 oncogenic pathway.

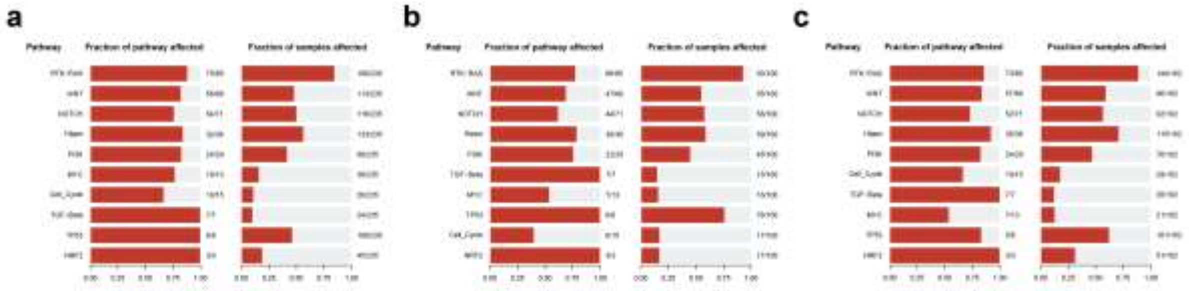

Supplementary Fig. S3 Expression level of prognostic genes in the TCGA–LUAD dataset. ***$P < 0.001$. TCGA–LUAD: The Cancer Genome Atlas–Lung Adenocarcinoma.

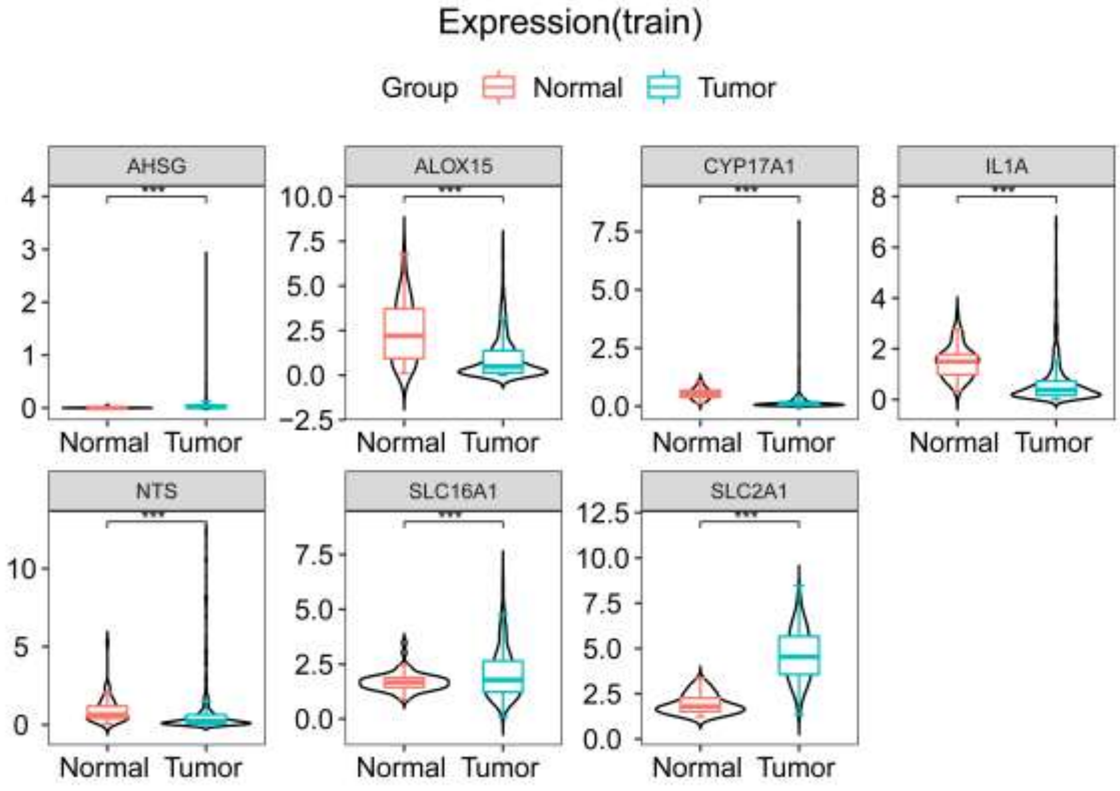

Supplementary Fig. S4 Expression level of prognostic genes in the GSE30219 dataset. ns represents not significant; **$P < 0.01$; ***$P < 0.001$.

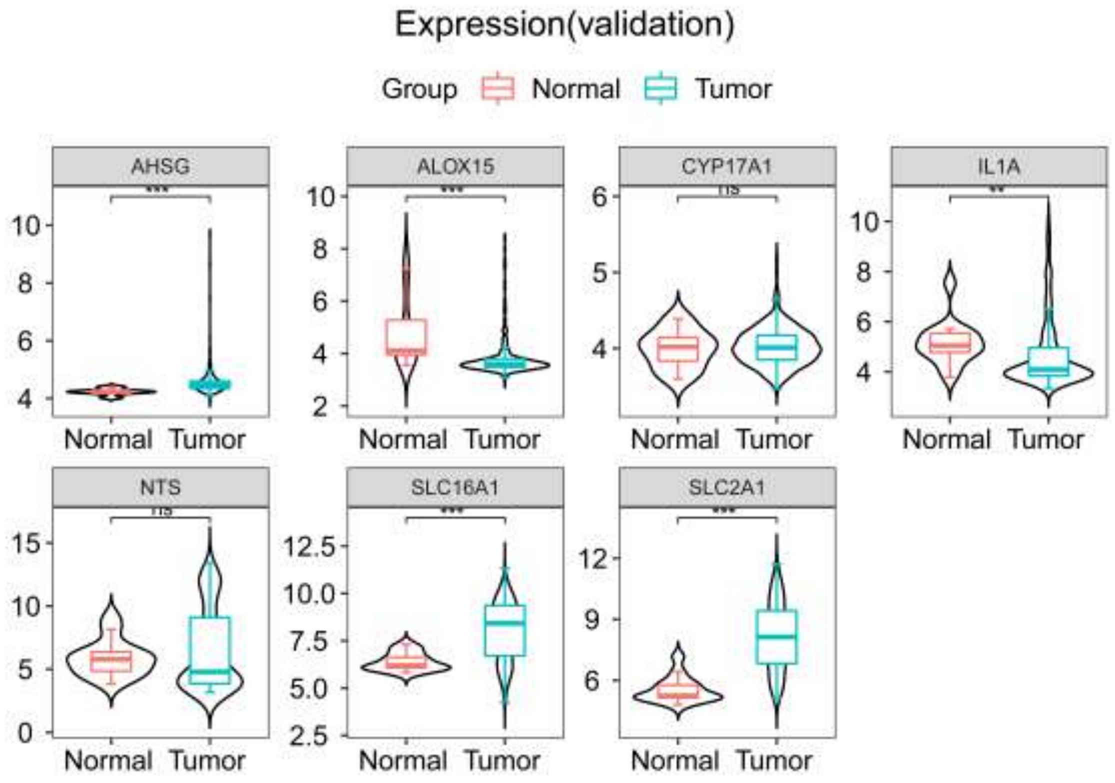

Supplementary Fig. S5 Correlation between risk scores and clinical indicators. (a) Differences in risk score expression across clinical indicator subgroups (age, gender, and T/N/M stages). ns represents not significant; $^{**}P < 0.01$. (b) Survival differences between high- and low-risk groups within clinical indicators.

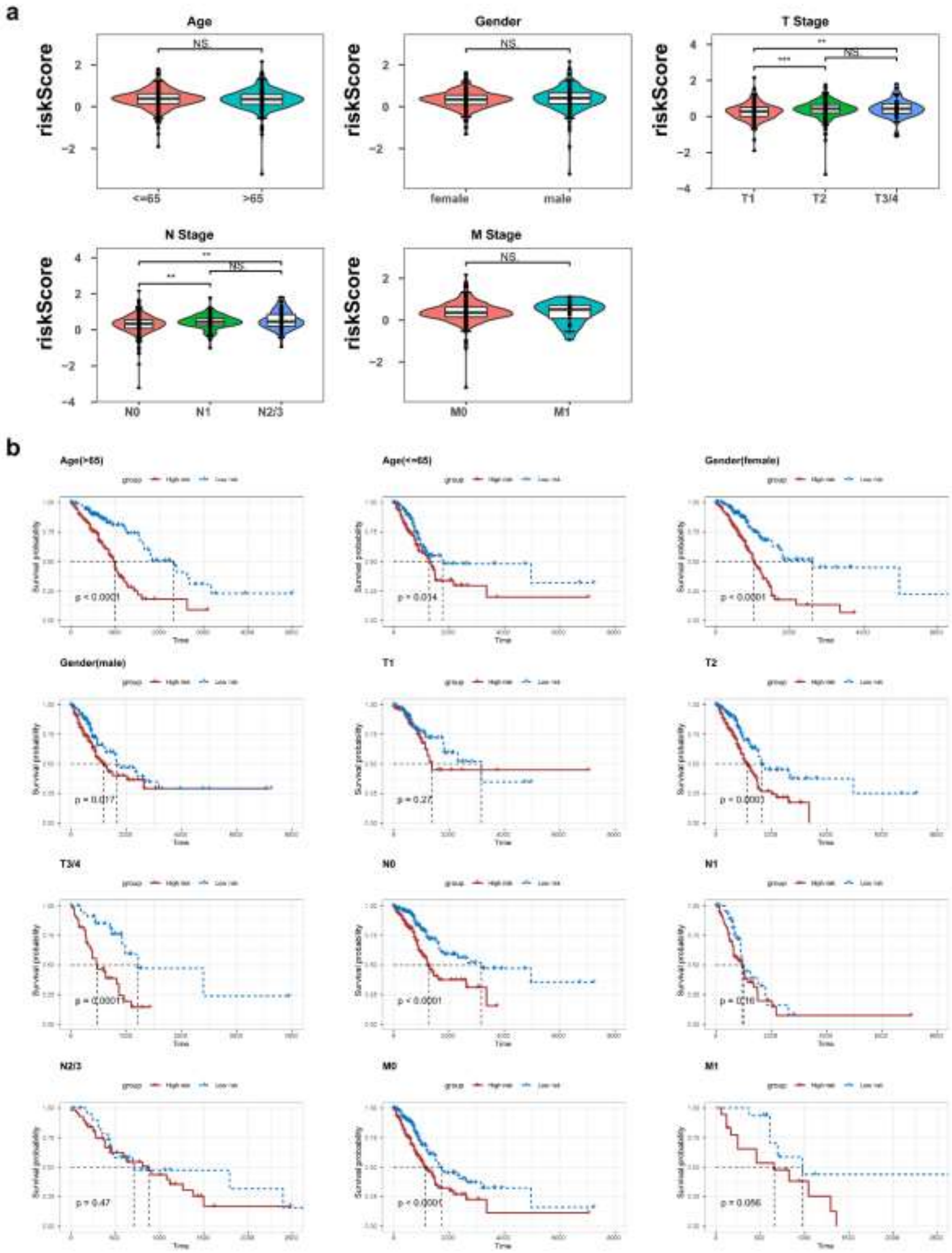

Supplementary Fig. S6 Differential expression analysis and functional enrichment of high- and low-risk groups. (a-b) Differential expression analysis and DEGs expression profiles between high- and low-risk groups. (c-d) GO and KEGG enrichment analyses of DEGs between high- and low-risk groups. DEGs: Differentially Expressed Genes, GO: Gene Ontology, KEGG: Kyoto Encyclopedia of Genes and Genomes.

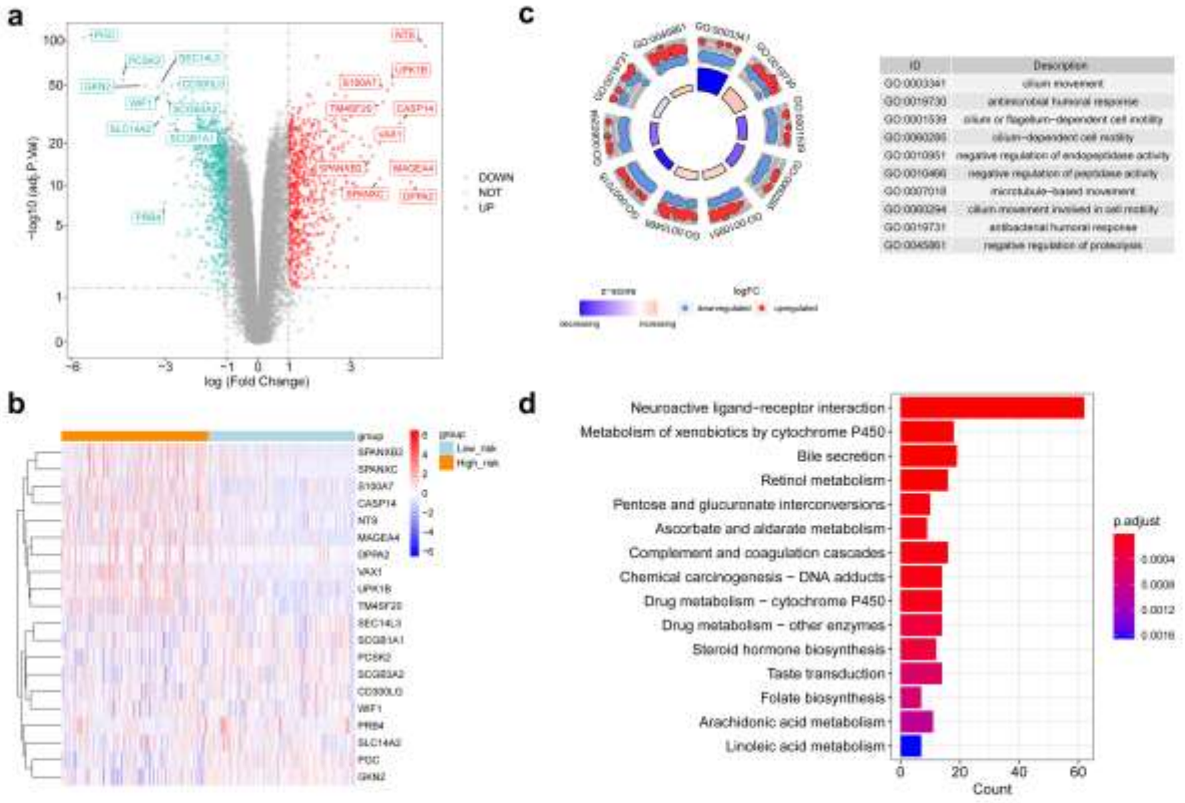

Supplementary Fig. S7 Analysis of drug sensitivity in high- and low-risk groups. (a) IC_{50} of drugs was lower in the high-risk group than in the low-risk group. (b) IC_{50} of drugs was higher in the high-risk group than in the low-risk group. ****$P < 0.001$.

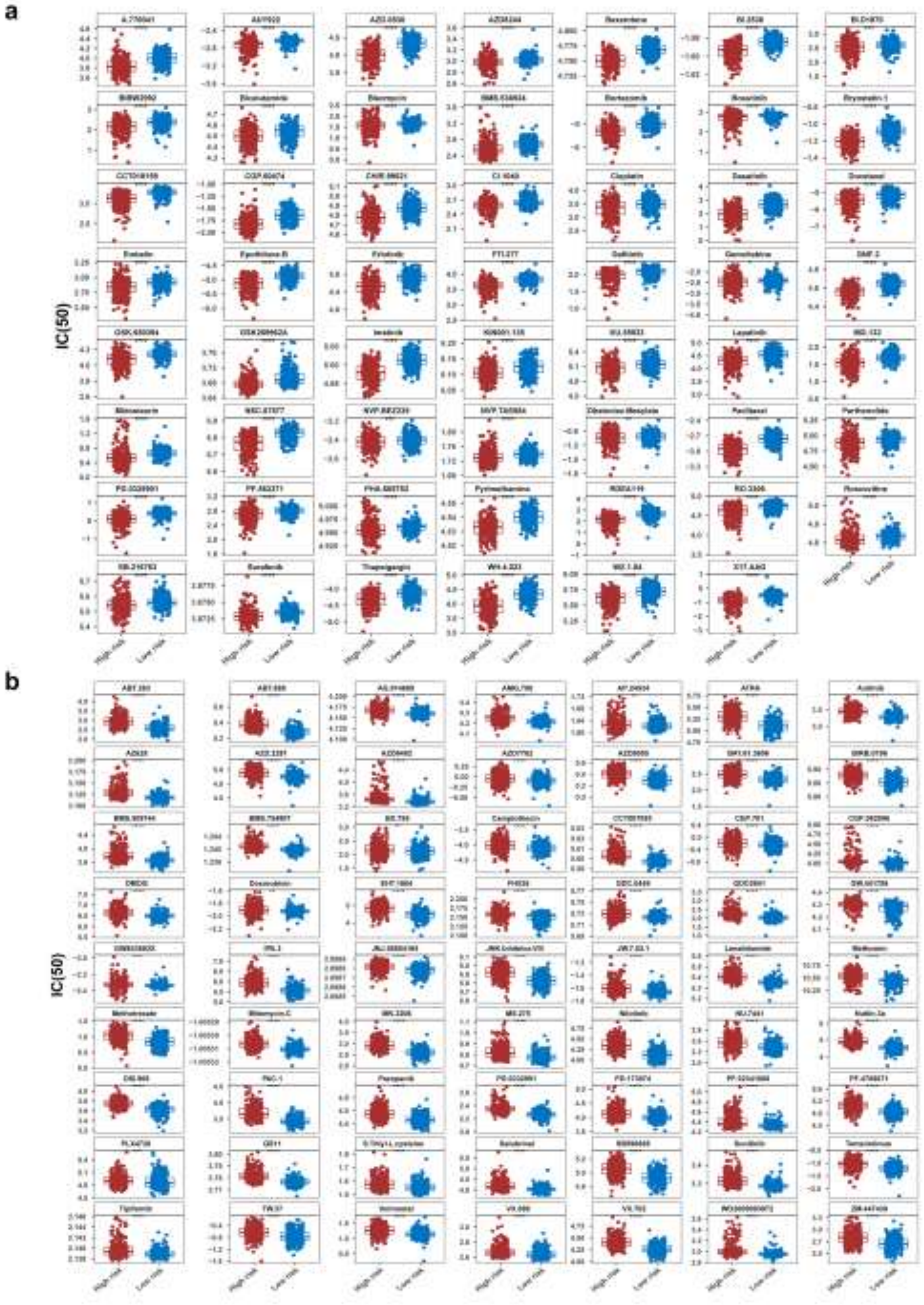

Supplement: Supplementary file 2 [file medi-105-e47270-s002.pdf]
